# Distinct effects of glucose and glucosamine on vascular endothelial and smooth muscle cells: Evidence for a protective role for glucosamine in atherosclerosis

**DOI:** 10.1186/1475-2840-4-16

**Published:** 2005-10-05

**Authors:** Wenlan Duan, Latha Paka, Sivaram Pillarisetti

**Affiliations:** 1 Reddy US therapeutics, 3065 Northwoods Circle, Norcross, GA 30071, USA; 2 Angion Biomedica, 350 Community Dr, Manhasset, NY 11030; 3 Department of Radiation Oncology, North Shore-Long Island Jewish Health System, 350 Community Dr, Manhasset, NY 11030, USA

## Abstract

Accelerated atherosclerosis is one of the major vascular complications of diabetes. Factors including hyperglycemia and hyperinsulinemia may contribute to accelerated vascular disease. Among the several mechanisms proposed to explain the link between hyperglycemia and vascular dysfunction is the hexosamine pathway, where glucose is converted to glucosamine. Although some animal experiments suggest that glucosamine may mediate insulin resistance, it is not clear whether glucosamine is the mediator of vascular complications associated with hyperglycemia. Several processes may contribute to diabetic atherosclerosis including decreased vascular heparin sulfate proteoglycans (HSPG), increased endothelial permeability and increased smooth muscle cell (SMC) proliferation. In this study, we determined the effects of glucose and glucosamine on endothelial cells and SMCs in vitro and on atherosclerosis in apoE null mice. Incubation of endothelial cells with glucosamine, but not glucose, significantly increased matrix HSPG (perlecan) containing heparin-like sequences. Increased HSPG in endothelial cells was associated with decreased protein transport across endothelial cell monolayers and decreased monocyte binding to subendothelial matrix. Glucose increased SMC proliferation, whereas glucosamine significantly inhibited SMC growth. The antiproliferative effect of glucosamine was mediated via induction of perlecan HSPG. We tested if glucosamine affects atherosclerosis development in apoE-null mice. Glucosamine significantly reduced the atherosclerotic lesion in aortic root. (P < 0.05) These data suggest that macrovascular disease associated with hyperglycemia is unlikely due to glucosamine. In fact, glucosamine by increasing HSPG showed atheroprotective effects.

## Introduction

Generalized vascular dysfunction including microvascular (nephropathy) and macrovascular (accelerated atherosclerosis) is a characteristic of diabetic complications [1-3]. Hyperglycemia by several mechanisms may contribute to increased atherosclerosis. Glucose can increase intracellular oxidative stress and generation of reactive oxygen species in endothelial cells [3]. This can result in activation of Redox sensitive transcription factors such as nuclear factor kappa B and inflammatory genes. Glucose can form adducts with proteins by non-enzymatic mechanisms leading to generation of glycated proteins and advanced glycation end products (AGE) [4,5]. In different animal models blocking AGE and its receptor RAGE reduced vascular disease [6]. Another pathway that has been postulated to play an important role in insulin resistance and potentially vascular complications is the hexosamine pathway [7,8]. In this pathway glucose is converted to glucosamine by the enzymatic actions of glutamine:fructose-6-phosphate amidotransferase (GFAT). In vitro in certain cell types glucosamine was shown to increase expression of growth factor TGFβ and PAI-1 [9,10]. Glucosamine is also the precursor for proteoglycan biosynthesis and increases proteoglycan production including heparan sulfate proteoglycan (HSPG) production in different cell types including vascular cells.

In vessel wall HSPG are produced by all cell types either as components of cell membrane (syndecan and glypican) or extracellular matrix (perlecan) [11]. Perlecan is the major HSPG of endothelial cells and SMC [12,13]. In atherosclerotic lesions the content of HSPG is reduced and studies show an inverse correlation between the amount of cholesterol in the lesion and the concentration of HS [14,15]. Thus, unlike chondroitin sulfate proteoglycans, HSPG is negatively correlated with atherosclerosis.

In this study we show that glucosamine and glucose have distinct effects on vascular HSPG and cell growth and glucosamine by virtue of its beneficial effects in vascular cells also reduces atherosclerosis in mice.

## Methods

### Materials

D (+) Glucosamine and D(+) glucose were purchased from Sigma Chemical Co. (St. Louis, MO). L- (4,5 ^3^H) Leucine, ^3^H-Thymidine, (^35^S) as sulfate in aqueous solutions and (^125^I) were from Amersham Life Science Corp (Arlington Heights, IL). Heparinase, heparitinase and chondroitinase ABC were purchased from Seikagaku America Inc (Bethesda, MD).

### Lipoproteins

LDL (d < 1.063), Lp(a) (d = 1.11) and HDL (d = 1.1–1.23) were isolated from fresh plasma by sequential ultracentrifugation. For some experiments ^125^I-LDL radio-iodinated using iodine monochloride [5] was used.

### Endothelial cells

Bovine aortic endothelial cells were isolated and cultured as described [20]. The cells (5–20 passages) were grown in minimal essential medium containing 10% FBS (Life-Technologies, Gaithersburg, MD).

### ^125^I-LDL transport

For ^125^I-LDL transport experiments, endothelial cells were grown in tissue culture inserts (Falcon-0.3 μm pore size) to facilitate its access to the upper (luminal) and lower (subendothelial) surface of endothelial cells. The barrier function of the endothelial cell monolayer was examined as previously reported [16], using [^3^H]dextran (average mol wt 150,000) and [^14^C]albumin. Transport of these molecules from the apical to the basolateral side of the monolayers was < 5%/h, a rate similar to that reported by others. At the conclusion of each LDL transport experiment, the monolayers were stained with 2% toluidene blue to verify the uniformity of the monolayer. On the day of the experiment, ^125^I-labeled LDL was added to the upper chamber and following incubation radioactivity in the lower-chamber medium and associated with the extracellular matrix was determined. The total amount of each lipoprotein transported across the monolayers (net transport) is the sum of these two measurements. To determine ^125^I-labeled LDL associated with the subendothelial cell matrix, the cells were incubated for 10 min with medium containing 50 U/ml of heparin (Elkins-Sinn, Inc., Cherry Hill, NJ)

### Metabolic labeling and preparation of subendothelial matrix (SEM)

Endothelial PG was radiolabeled with (^35^S) sulfate along with the indicated doses of glucosamine for 12–16 h. Cellular PG were assessed by removing the cells with Triton X-100/NH_4_OH. Endothelial cells were grown in 24 or 48 well plates (Falcon: Beckton Dickinson, Lincoln Park, NJ). Subendothelial matrix (SEM) was prepared as described [17]. Briefly, confluent monolayers of endothelial cells were washed three times with PBS and incubated for 10 min in a solution containing 0.1% TritonX-100 and 20 mM NH_4_OH at room temperature. Detached cells were removed by washing four times with PBS. This procedure leaves the intact matrix attached to the surface of the well. Matrix PG was extracted with 6 M guanidine HCl for 4 h at 4°C. For enzyme treatments, SEM was incubated with a mixture of heparinase and heparitinase (1 U/ml each) or chondroitinase ABC (1–2 U/ml) for 3 h at 37°C.

### Glycosaminoglycan (GAG) size estimation

To prepare GAG chains, 1-ml aliquots of purified cell surface PG were treated with 100 μl of 10 N NaOH for 18 h at 26°C with constant shaking and then neutralized with 10 N HCl [18]. To remove core proteins, the samples were adjusted to 7 M urea and loaded on 1-ml DEAE mini column that was previously equilibrated with 3 bed volumes of 7 M urea, 0.1% Triton X-100, 0.2 M NaCl in 0.05 M Tris, pH7.2. Peak fractions were pooled and dialyzed against 10 mM Tris and 0.1% Triton X-100 to remove urea. To determine GAG size, 0.6 ml of the above protein free GAG were chromatographed on Sephacryl-200 (Pharmacia-Biotech) gel-filtration column that was previously calibrated with known molecular weight standards using 0.2 M NaCl as an eluate.

### Monocytes and adhesion assay

THP-1 cells were purchased from the American Type Culture Collection (Rockville, MD) and were grown in RPMI 1640 (Gibco-Laboratories, Grand Island, NY) containing 10% fetal bovine serum (Gemini Bioproducts Inc., Calabasas, CA).

Adhesion assay was done as described previously [19]. Monocytes were incubated in leucine-free DMEM-BSA medium before labeling. 100 μCi of (^3^H) leucine was added to 1 × 10^7 ^cells and incubated for another 2 h under cell culture conditions. The labeled cells were centrifuged at 800 rpm for 5 min to remove the unincorporated label. The cells were washed three times and re-suspended in DMEM-BSA and then added to endothelial cell monolayers or to SEM in 24 well plates (24 × 10^5 ^cells/well). Binding was performed for 2 h at 37°C. Unbound monocytes were removed by washing four times with DMEM-BSA and the bound radioactivity was extracted with 0.5 N NaOH for 1 h at 37°C and then counted.

### SMC proliferation

Rat aortic SMCs were cultured as previously described [20] in basal medium supplemented with growth factors, bFGF, hEGF and 5% fetal bovine serum (Clonetics Corp, San Diego, CA). SMCs were plated in low density (9 × 10^4 ^cells/ well) in 6 or 12 well plates and cultured in the presence or absence of 30 mM glucose or 2.5 mM glucosamine for three days. The cells were then trypsinized and an aliquot of trypsinate was counted for the final cell number with hemacytometer. Net growth was assessed by subtracting the final cell number from the initial cell number.

In other experiments, cells grown under above conditions were labeled with (^3^H)-thymidine (5 μCi/ml) for 6 h and the cells were washed four times with DMEM-BSA to remove unincorporated label. The cells were then lysed by 0.5 N NaOH and the thymidine incorporation into the DNA was assessed.

### Animal studies

#### Sulfate incorporation in tissues of mice

To determine if glucosamine increases HS in vivo (determined by ^35^SO_4 _incorporation), C57Bl/6J mice from Jackson Laboratories (Bar Harbor, Maine) (8 weeks old, three controls and three glucosamine) were given saline or saline containing 5 mg/kg of glucosamine intraperitoneally for 3 days. On the day of experiment, mice were given 100 μCi of ^35^SO_4 _in 100 μl of saline. Mice were sacrificed after 4 h, tissues were perfused with PBS and liver and heart together with proximal aorta, were removed. Tissues were homogenized with polytron for 30 sec in ice cold HEPES buffer containing 4 M urea, 0.5% CHAPS, 0.5 M NaCl, 1 mM each of PMSF, benzamidine-hydrochloride and 5 μg/ml of leupeptin. Tissue homogenates were centrifuged (14000 rpm, 20 min) and the supernatants were dialyzed extensively against PBS to remove low molecular weight free sulfate. Aliquots of dialyzed supernatants were precipitated with 3 volumes of alcohol and counted and the radioactivity was expressed per mg of tissue protein.

#### Glucosamine effects on atherosclerosis

Male apoE-/- mice on C57BL/6J background were purchased from Jackson Laboratories (Bar Harbor, Maine). Mice were housed at 25°C on a 12 h light-dark cycle and were fed on a chow diet and water ad libitum throughout the study. At four weeks of age, they were randomly divided into vehicle (n = 7) and glucosamine treated (n = 8) groups. Glucosamine were administered intraperitoneally once a day (5 mg/kg). The study was terminated at 12 weeks of age. Mice were euthanized by CO_2 _and exanguination. Blood samples were collected for glucose and lipid assay by Colorimetric assays (Sigma Diagnostics). Aortic roots were snap frozen in (optimal cutting temperature) OCT for lesion evaluation by Oil-Red-O staining.

## Results

### Glucosamine but not glucose increases ^35^SO_4 _incorporation into endothelial HSPG

**Figure 1:**
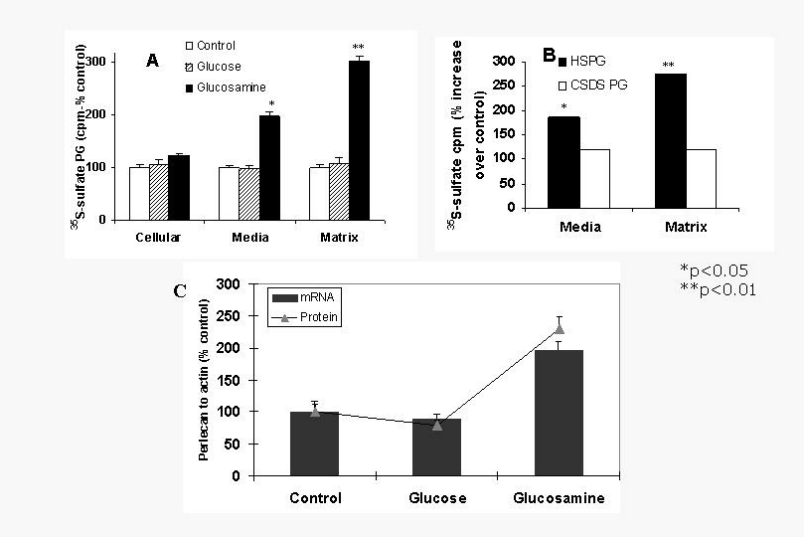
A. Glucosamine treatment increases ^35^SO_4 _incorporation into PG. Endothelial cells were labeled with ^35^S sulfate (25 μCi/ml) in medium alone (Control) or medium containing 30 mM glucose or 2.5 mM glucosamine 16 h. Radioactivity associated with proteoglycans (PG) from cells, media and extracellular matrix was determined. Values are expressed as mean ± SD B. Glucosamine mediated increase in PG is mostly in heparan sulfate proteoglycans (HSPG). Media and matrix PG prepared from control and glucosamine treated endothelial cells were treated with chondroitinase ABC for 4 h at 37°C and undigested PG were precipitated and determined as HSPG. To determine if the undigested glycosaminoglycan (GAG) is HS, aliquots were subjected to low pH nitrous acid digestion. 1C. Glucose and glucosamine effects on perlecan mRNA (Real time PCR – 1C bar) and protein (immunoprecipitated protein in media 1C line) p < 0.01.

**Figure 2:**
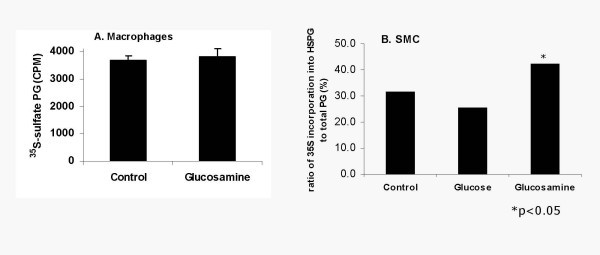
Glucosamine effects on macrophage (A) and smooth muscle cells (SMC) (B) PG. THP-1 human monocyte-macrophages or confluent monolayers of rat aortic SMC were labeled with ^35^SO_4 _in medium or medium containing 2.5 mM glucosamine or medium containing 30 mM glucose (for SMC) for 16 hours. Total PG and CS/DS PG and HSPG were determined as described in Figure 1B. (data not mentioned in the Result)

To determine whether glucosamine increases endothelial HS production, aortic endothelial cell proteoglycans were labeled with ^35^S sulfate. Glucosamine treatment increased ^35^SO_4 _incorporation into the media PG by 2 fold and into matrix PG by 3 fold (Figure 1A). Addition of glucose (30 mM) to the medium did not affect PG production. Enzymatic analysis showed that the increase was found to be exclusively in HSPG and glucosamine treatment did not affect CS/DS PG in endothelial cells (Figure 1B). Perlecan is the major HSPG secreted by endothelial cells, we therefore tested if the increase was in perlecan. Real time PCR analysis showed a 1.9 fold increase in perlecan mRNA (Figure 1C bars) and consistent with this immunoprecipitation analysis showed that media from glucosamine treated cells contain 2.3 fold perlecan in medium (Figure 1C line). Thus these data suggest that glucosamine primarily increased endothelial cell perlecan. Highly sulfated blocks of HS are referred to as heparin-like HS, which confer several biological properties to HS. To identify heparin-like HS GAG, matrix HSPG were subjected to heparitinase digestion, followed by low pH nitrous acid digestion [18,21]. Heparitinase-resistant and nitrous acid-sensitive HS was increased by two fold (2300 cpm in control versus 4550 cpm in glucosamine treated) suggesting that glucosamine treatment of endothelial cells increased heparin-like HS. Glucose or glucosamine did not significantly increase macrophage PG but glucosamine showed a moderate increase in SMC HSPG (Figure 2).

### Glucosamine treatment improved endothelial barrier function

**Figure 3:**
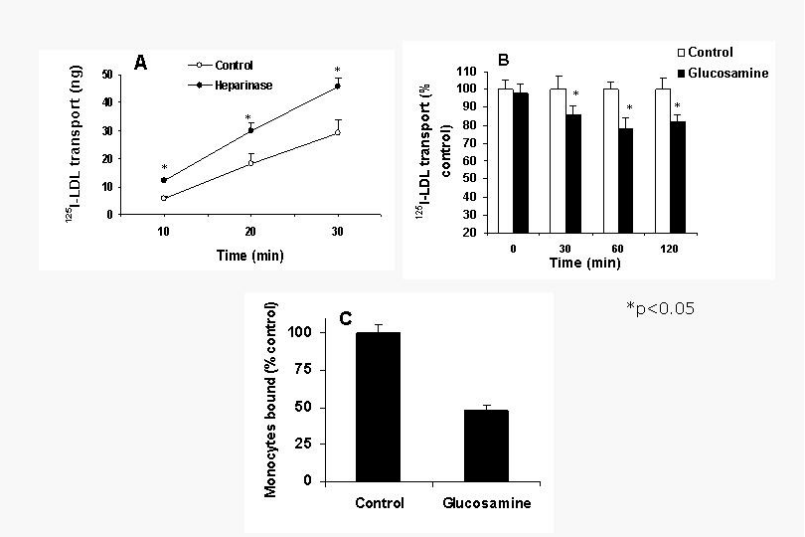
A. HSPG modulate LDL transport across EC monolayers. Endothelial cells were grown to confluence in tissue culture inserts (Falcon, 0.3 μm pore size) in 24 well plates to facilitate its access to the upper (luminal) and lower (subendothelial) surface of endothelial cells. The cells were incubated with medium alone (control) or medium containing 1 unit/ml each of heparinase and heparitinase in the bottom chamber for 2 h at 37°C. ^125^I-LDL was then added to the cells in the upper chamber and the ^125^I-LDL appeared in the media from the lower chamber was counted. Values represent Mean ± SD of triplicate measurements. B. Glucosamine treatment decreases LDL transport. Endothelial cells on tissue culture inserts were incubated with medium alone or medium containing 2.5 mM glucosamine for 16 h. ^125^I-LDL transport was then determined as described above. Figure 3C. Monocyte adhesion to glucosamine treated endothelial cells decreases. Endothelial cells were grown to confluence in 24 well tissue culture plates. Cells were then incubated in medium or medium with glucosamine for 16 h. Subendothelial matrix was prepared from control and glucosamine treated endothelial cells and incubated with (^3^H)leucine labeled THP-1 monocytes for 2 h. Unbound monocytes were washed four times with DMEM-BSA and the bound radioactivity was determined.

Subendothelial matrix HSPG is thought to play a key role in endothelial barrier function, however, whether decreased HSPG increases lipoprotein transport across endothelial monolayers is not known. We first tested whether removal of HSPG increases LDL transport across endothelium. Removal of HSPG by heparinase treatment led to a 2.1 fold increase in ^125^I-LDL transport at 10 min. (Figure 3A). At 15 min and 30 min the increase in LDL transport was 57% and 36% higher than controls. Conversely, after glucosamine treatment, ^125^I-LDL transport across the EC monolayers decreased by 15–22% at different time points in glucosamine treated cells (Figure 3B).

### Monocyte binding to matrix is decreased in glucosamine treated endothelial cells

We previously showed that removal of HSPG increases monocyte retention in the subendothelial matrix [19]. We determined THP-1 monocyte to the subendothelial matrix prepared from control, glucose and glucosamine-treated cells. Monocyte binding to the glucosamine stimulated endothelial cells was decreased by 52% compared to non-stimulated cells (Fig. 3C). In contrast monocyte binding to glucose-treated endothelial cells was slightly increased similar to previous observations (20%, not shown ref [22]).

### Glucose and Glucosamine effects on PG in SMC- Glucosamine but not glucose treatment decreases SMC proliferation

**Figure 4:**
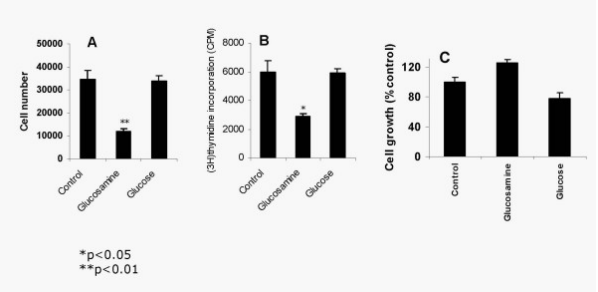
Glucosamine treatment decreases the growth of SMC (A and B) but not endothelial cell (C) proliferation. Sub-confluent SMC (9 × 10^4^/well) were incubated in growth medium or growth medium containing 2.5 mM glucosamine or 30 mM glucose for 24–48 h and cell growth was determined. (A) Initial and final cell (SMC) number was counted and net growth was determined. (B) SMC were labeled with ^3^H-Thymidine and its incorporation into the DNA was assessed. (C) Endothelial cell growth was determined by thymidine incorporation into DNA.

**Figure 5:**
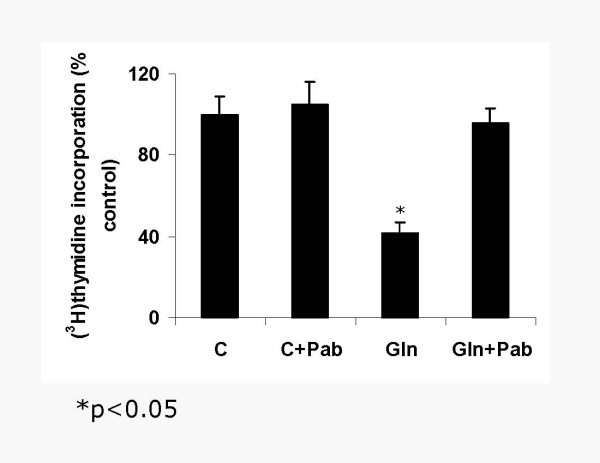
The antiproliferative effect of glucosamine requires perlecan: SMC proliferation was carried out as described in Figure 4 with or without anti-perlecan antibody (10 μg/ml) in the presence or absence of glucosamine.

We next determined the effects of glucose and glucosamine on SMC proliferation. Sub-confluent SMC were incubated in media containing 30 mM glucose or 2.5 mM glucosamine for 48 h and net growth was determined. Glucose treatment did not alter SMC growth as assessed by cell number (Figure 4A) or thymidine incorporation (Figure 4B). In contrast, the cell number and ^3^H thymidine incorporation into the DNA was decreased by 60% by glucosamine treatment (4A and B). The major HSPG secreted by SMC is perlecan, which was known to negatively correlate with SMC growth [11]. Immunoprecipitation analysis showed a 2.5 fold increase in perlecan protein in SMC treated with glucosamine (not shown). We next tested whether perlecan mediates the antiproliferative effect of glucosamine on SMC. Addition of an anti-perlecan antibody completely abolished the antiproliferative effect of glucosamine on SMC proliferation (Figure 5). These results suggest that glucosamine treatment increases perlecan production, which in turn modulates SMC growth in proliferating cells. Consistent with a perlecan-based mechanism, glucosamine did not inhibit endothelial cell growth (Figure 4C)

### Glucosamine increases HS and reduces atherosclerotic lesion of aortic root in vivo

**Figure 6:**
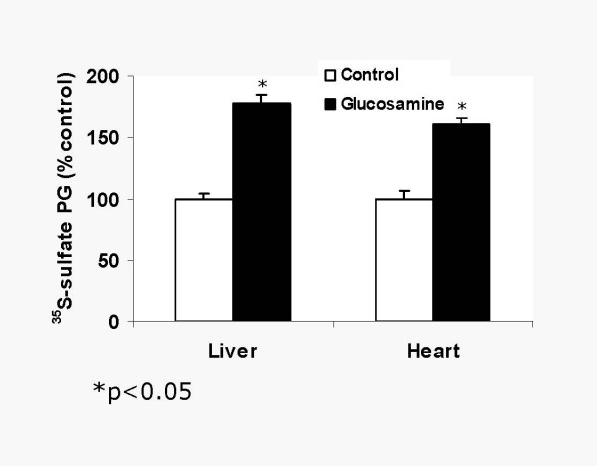
Glucosamine increases ^35^SO_4 _incorporation in vivo. To determine if glucosamine can increase tissue PG, mice were injected with glucosamine (intraperitoneal, 100 ul containing 5 mg, every other day for 3 days). On the day of the experiment ^35^SO_4 _was injected intravenously and mice were sacrificed after 4 h. Following perfusion with saline, liver, and heart were removed and homogenized in phosphate buffered saline containing 1 mM PMSF, 1 mM benzamidine and 0.5% CHAPS. PG were precipitated with 3 volumes of 100% alcohol. ^35^S-cpm in the precipitate was determined.

**Figure 7:**
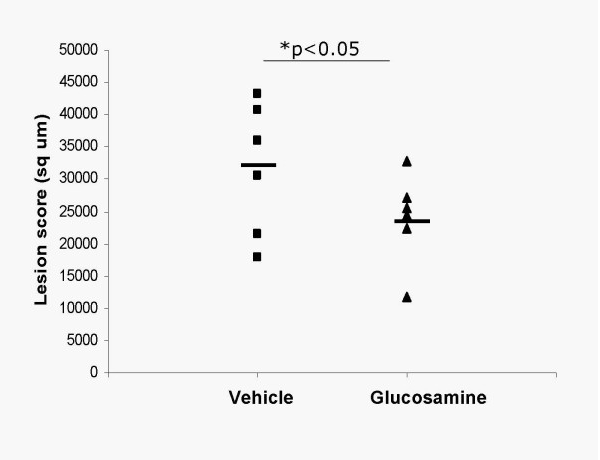
Glucosamine reduces atherosclerotic lesion of aortic root in apoE null mice. apoE null mice were treated with glucosamine intraperitoneally at 5 mg/kg for 2 months from 4 weeks to 12 weeks of age. At the end of the study, mice were euthanized. Aortic roots were collected and snap frozen in OCT for lesion evaluation by Oil-Red-O staining.

We tested glucosamine effects on HSPG and atherosclerosis. When given intraperitoneally (5 mg/kg once a day for 3 days) glucosamine increased ^35^S sulfate incorporation into HSPG in liver (by 61%) and heart (by 82%) (Figure 6). To determine the effects of glucosamine on atherosclerosis, apoE null mice were treated with glucosamine at 5 mg/kg for 2 months. Plasma glucose and total cholesterol was not affected by glucosamine (not shown). Oil Red-O staining revealed 30% reduction in lesions in glucosamine treated group (p < 0.05) (Figure 7). These data suggest that macrovascular disease associated with hyperglycemia is unlikely due to glucosamine. In fact, glucosamine by inducing HSPG showed atheroprotective effects.

## Discussion

Glucosamine is a precursor of GAG biosynthesis. In cells glucosamine is produced from glucose by the hexosamine pathway in a reaction requiring fructose 6-phosphate and glutamine and catalyzed by the enzyme glutamine:fructose-6 phosphate amidotransferase [7,8]. About 1–2% of the incoming glucose enters this pathway. Glucosamine is primarily used for protein glycosylation and GAG synthesis [23]. Although cells can synthesize glucosamine, exogenous glucosamine can also be taken up and converted to its 6-phosphate derivative, which can then be utilized for HSPG synthesis. Current studies show that glucose and glucosamine have distinct effects on vascular HSPG and cell proliferation. Unlike glucose, glucosamine increased matrix HSPG (perlecan) production both in endothelial cells and SMC. Glucosamine increased sulfate incorporation specifically into HSPG.

Several previous studies suggested a role for glucosamine in the development of insulin resistance [8,24]. Based on several in vitro studies glucosamine was also suggested to be a player in mediating the vascular complications [7,9]. These observation received great attention because glucosamine is frequently used by patients with osteoarthritis [25]. However, recent studies show that in humans at doses used by arthritis patients glucosamine does not appear to induce insulin resistance [26].

Loss of endothelial HS has been postulated to lead to several pathological events, in particular to events related to atherosclerosis [13,27]. Agents that decrease endothelial HSPG include, lipopolysaccharide, TNF-alpha [28], homocysteine [29], lysolecithin and oxidized LDL [30]. Thus, decrease in HS may be a general inflammatory reaction.

In atherosclerosis, CS/DS PG positively correlated with lesion progression [14,15]. Thus, glucosamine treatment not only increased athero-protective HSPG but also decreased (in SMC, figure 2B) or did not affect (in endothelial cell, figure 1B) atherogenic CS/DS PG. Glucosamine also increased the amount of heparin-like HS (oligosaccharide sequences that are resistant to heparitinase digestion but sensitive to heparinase and low pH nitrous acid) in endothelial cells. Subendothelial HSPG (perlecan) contains substantial amounts of these sequences [31]. In our experiments, glucosamine primarily increased extracellular HSPG (in media and in the matrix).

Glucosamine treatment also inhibited SMC proliferation. The extent of this inhibition was much greater than that of other antiproliferative substances such as apoE, nitric oxide and TGF-β (not shown). The antiproliferative effect of glucosamine is most likely due to increased HSPG production in media. It has been well documented that while cell surface HSPG are required for growth factor activity (as co-receptors) exogenous HSPG are a potent inhibitors of SMC proliferation [32,33]. Perlecan is the major HSPG secreted by SMC and it negatively correlates with SMC proliferation [34]. In the present studies an anti-perlecan antibody completely blocked glucosamine antiproliferative effect. These data suggest that glucosamine inhibits SMC proliferation by increasing media perlecan. These data also show that the SMC growth inhibition by glucosamine is not due to general cell toxicity. Among the vascular cells only SMC, but not endothelial cells and macrophages, are sensitive to HSPG inhibition. Consistent with this glucosamine did not inhibit growth of endothelial cells.

How glucosamine increased perlecan production is not clear. Our data suggest that glucosamine increased HS GAG content but not chain length. Because perlecan contains only three HS chains per core protein, it is conceivable that more perlecan core protein is associated with HS chains facilitating its secretion. Alternatively, glucosamine may have induced perlecan expression. Glucosamine is also utilized for glycosylation of proteins including certain transcription factors. Glycosylation state of the transcription factors affects their activity [35]. Thus, it is conceivable that glucosamine treatment increased the glycosylation of transcription factors involved in perlecan expression. Glucosamine was also shown to induce growth factor expression [36-38], such as TGFβ, which can stimulate perlecan [39]. However, it should be noted that in these studies, in contrast to the present experiments, high concentrations of glucose had the same effect as that of glucosamine. Nevertheless, if this occurs, since perlecan antibody blocked the antiproliferative effect of glucosamine, these data raise the possibility that the antiproliferative effects of TGFβ are mediated by perlecan.

Our data also showed that glucosamine administration to mice increased ^35^SO_4 _incorporation in tissues. Glucosamine is taken by cells via the glucose transporters and is generally absent in circulating plasma [40]. A dose of 5 mg/kg of glucosamine for three days increased ^35^SO_4 _incorporation into the liver by 82% and into hearts (containing proximal aorta) by 61%. It remains to be determined whether this increase is specifically in HSPG. The in vivo effect of glucosamine was tested in one other study. Because CS and DS PG are thought to mediate lipoprotein retention and therefore, atherogenic. Recent in vitro studies showed that glucosamine induced proteoglycans have reduced binding to LDL therefore less atherogenic [41]. An earlier study looked at the effect of glucosamine on plasma and aortic cholesterol in rabbits [42]. Surprisingly, this study found a two fold decrease in cholesterol/unit-wet weight of aorta. However, our data showing that glucosamine increases only HSPG but not CS/DS may explain why lipoprotein accumulation is decreased. The ability of glucosamine to inhibit atherogenesis has recently been postulated [43] and has been demonstrated in our apoE null mice study. This occurred without changes in plasma glucose and lipids. Taken together with our present data, studying the effects of increased HSPG on atherosclerosis appears to be feasible in mice.

In summary, our data show that glucosamine increases HSPG production in vascular cells and ^35^SO_4 _incorporation into tissue. Atherogenic PG, like CS/DS PG were not increased. By increasing HSPG, glucosamine reduced atherogenic events including lipoprotein transport, monocyte retention and SMC proliferation, combined with it protective effect on atherosclerotic lesion in apoE null mice, raising the possibility that it is a potential anti-atherogenic agent.

## Abbreviations

HSPG – heparan sulfate proteoglycans, PG-proteoglycan, LDL-low density lipoprotein, SMC – smooth muscle cell, GAG – glycosaminoglycan

## Competing interests

The author(s) declare that they have no competing interests.

## Authors' contributions

WD performed in vivo studies and LP performed in vitro studies. SP designed, supervised the study and provided support.
